# Protection against Doxorubicin-Induced Cytotoxicity by Geniposide Involves AMPK*α* Signaling Pathway

**DOI:** 10.1155/2019/7901735

**Published:** 2019-06-26

**Authors:** Yan-Yan Meng, Yu-Pei Yuan, Xin Zhang, Chun-Yan Kong, Peng Song, Zhen-Guo Ma, Qi-Zhu Tang

**Affiliations:** ^1^Department of Cardiology, Renmin Hospital of Wuhan University, Wuhan 430060, China; ^2^Cardiovascular Research Institute of Wuhan University, Wuhan 430060, China; ^3^Hubei Key Laboratory of Cardiology, Wuhan 430060, China

## Abstract

Oxidative stress and cardiomyocyte apoptosis play critical roles in the development of doxorubicin- (DOX-) induced cardiotoxicity. Our previous study found that geniposide (GE) could inhibit cardiac oxidative stress and apoptosis of cardiomyocytes but its role in DOX-induced heart injury remains unknown. Our study is aimed at investigating whether GE could protect against DOX-induced heart injury. The mice were subjected to a single intraperitoneal injection of DOX (15 mg/kg) to induce cardiomyopathy model. To explore the protective effects, GE was orally given for 10 days. The morphological examination and biochemical analysis were used to evaluate the effects of GE. H9C2 cells were used to verify the protective role of GE in vitro. GE treatment alleviated heart dysfunction and attenuated cardiac oxidative stress and cell loss induced by DOX in vivo and in vitro. GE could activate AMP-activated protein kinase *α* (AMPK*α*) in vivo and in vitro. Moreover, inhibition of AMPK*α* could abolish the protective effects of GE against DOX-induced oxidative stress and apoptosis. GE could protect against DOX-induced heart injury via activation of AMPK*α*. GE has therapeutic potential for the treatment of DOX cardiotoxicity.

## 1. Introduction

Doxorubicin (DOX) is an anthracycline that has been used to treat cancer for several decades and has potent broad-spectrum antineoplastic activity [1]. It has been used for the treatment of a variety of neoplasms including leukemia, lymphomas, thyroid cancer, ovarian cancer, and breast cancer [2–4]. However, its clinical application is largely limited by high incidence of irreversible side effects [5, 6]. Notably, the dose-dependent cardiotoxicity is the most dangerous complication of DOX which could eventually lead to fatal heart failure [7]. The mechanism involved in doxorubicin-induced cardiotoxicity is complex and multifactorial; nevertheless, accumulating evidences suggest that oxidative stress and apoptosis play pivotal roles [8, 9].

Cardiac tissue is especially vulnerable to free radicals induced by DOX because the hearts had a lot of mitochondria and cardiac antioxidant enzymes were relatively lower compared with other organs [10]. Previous studies have shown that increased levels of cardiac reactive oxygen species (ROS) were observed during the development of DOX-induced cardiomyopathy [11]. Excessive ROS accumulation caused lipid peroxidation, structural changes of biological macromolecules, and eventually result in cell apoptosis. Therefore, finding molecules that could inhibit oxidative stress and cardiomyocyte apoptosis would be of great significance for the prevention of DOX-related cardiac injury.

AMP-activated protein kinase *α* (AMPK*α*) plays a critical role in the process of various heart diseases [12–15]. It has been reported that the activation of AMPK*α* via pharmacological approaches prevented pressure overload or obesity-induced cardiac remodeling in mice [16–18]. The results from our and other labs demonstrated that activation of AMPK*α* could attenuate high glucose-induced oxidative stress and cell apoptosis [19]. Hence, finding a positive regulator of AMPK*α* would be of great significance for DOX-induced cardiotoxicity.

Geniposide (GE), an iridoid glycoside derived from the gardenia plant, has a series of pharmacological effects, including antioxidative stress, antiapoptosis, anti-inflammatory, anti-ER stress, and other pharmacological effects [20–22]. Our previous study demonstrated that GE could attenuate the development of pressure overload-induced cardiac hypertrophy and prevent obesity-related cardiac complications via activation of AMPK*α* [16, 17]. However, it still remains unclear whether GE could defend against DOX-induced cardiotoxicity via AMPK*α* and the precise mechanisms. In the present study, we have found that GE improves cardiac function via the activation of AMPK*α* in DOX-induced cardiac injury.

## 2. Materials and Methods

### 2.1. Reagents

Geniposide (GE) was purchased from Shanghai Winherb Medical Science Co. (Shanghai, China). DOX (D1515) was purchased from Sigma-Aldrich (St. Louis, MO, USA). The purity of GE and DOX was above 98% as determined by HPLC analysis. Antibodies against T-AMPK*α* (#2603P, 1 : 1000), P-AMPK*α* (#2535, 1 : 1000), T-acetyl-CoA carboxylase (ACC, #3676, 1 : 1000), P-ACC (#3661S, 1 : 1000), Bcl-2 (#2870, 1 : 1000), Bax (#2772, 1 : 1000), cleaved caspase-3 (C-Caspase3, #9661, 1 : 1000), glyceraldehyde 3-phosphate dehydrogenase (GAPDH, #2118, 1 : 1000), and T-AMPK*α*2 (#2757, 1 : 1000) were purchased from Cell Signaling Technology (Boston, MA, USA). The primary antibodies of nuclear factor erythroid-derived-like 2 (Nrf2, #ab31163, 1 : 1000), superoxide dismutase-1 (SOD1, #ab16831, 1 : 1000), and superoxide dismutase-2 (SOD2, #ab13533, 1 : 1000) were purchased from Abcam (Cambridge, UK). The cell counting kit-8 was purchased from Dōjindo Laboratories (Kumamoto, Japan). The bicinchoninic acid (BCA) protein assay kit was purchased from Pierce (Rockford, IL, USA).

### 2.2. Animals and Treatments

All animal experimental procedures were approved by the Guidelines for Animal Care and Use Committee of Renmin Hospital of Wuhan University, which is also in agreement with the Guidelines for the Care and Use of Laboratory Animals published by the United States National Institutes of Health (NIH Publication, revised 2011). Male C57BL/6 mice (8 to 10 weeks old; body weight: 25.5 ± 2 g) were obtained from the Institute of Laboratory Animal Science, Chinese Academy of Medical Sciences (Beijing, China). These mice were allowed free access to food and drinking water. They were fed in a house which the temperature (20-25°C) and humidity (50 ± 5%) were under strict control and the light was kept at a 12 h light-dark cycle. These mice were divided into four groups (*n* = 10 per group): saline group, DOX group, DOX+GE (25 mg/kg) group, and DOX+GE (50 mg/kg) group, by a random number table. DOX was dissolved in normal saline. Mice in the DOX and DOX+GE groups were subjected to a single intraperitoneal injection of DOX (15 mg/kg). Correspondingly, equal volume of saline was given to the control mice according to our previous study [23]. Mice in the DOX+GE groups were orally given a dose of GE (25 or 50 mg/kg, 9:00 am) dissolved in sterile saline every day for 10 days beginning from three days before DOX injection. To clarify whether the cardiac protective effects of GE on DOX-induced cardiotoxicity were mediated by AMPK*α*, we used AMPK*α*2 KO mice. The source of AMPK*α*2 knockout mice was described in our previous article [16, 17]. Seven days later, the mice were sacrificed with an overdose of sodium pentobarbital (200 mg/kg; i.p.) to harvest the hearts and calculate the ratios, like heart weight (HW)/body weight (BW) and HW/tibia length (TL).

### 2.3. Echocardiography and Hemodynamics

Echocardiography and hemodynamics were performed as described previously [24, 25]. Briefly, a MyLab 30CV ultrasound (Esaote SpA, Genoa, Italy) with a 10 MHz linear array ultrasound transducer was used to obtain M-mode images at the papillary muscle level for measurement of systolic and diastolic function.

For the hemodynamic analysis, a 1.4-French Millar catheter transducer (SPR-839; Millar Instruments, Houston, TX) was inserted into the left ventricle via the right carotid artery to obtain invasive hemodynamic measurements. PVAN data analysis software was used to analyze all the data.

### 2.4. Western Blot

Proteins were extracted from heart tissues or cells with RIPA lysis buffer (Invitrogen, Carlsbad, CA, USA) on ice. Then, proteins were separated on 10% SDS-PAGE and transferred to polyvinylidene fluoride (PVDF) membranes (Millipore). Next, the membranes were blocked with 5% skim milk in Tris-buffered saline-Tween (TBST) for 1 h. Afterwards, the PVDF membranes were incubated with the indicated primary antibodies overnight at 4°C and with secondary antibodies for another 1 h at room temperature. Finally, these membranes were scanned, and the total protein levels were normalized to GAPDH. Phosphorylation was normalized to the matched total protein.

### 2.5. Total RNA Extraction and Quantitative Real-Time PCR

Trizol reagents were used to extract total RNA from heart tissue and cells. Reverse transcriptional reactions were performed using the Transcriptor First Strand cDNA Synthesis Kit (Roche Applied Science, USA). The target genes were amplified with the LightCycler 480 SYBR Green Master Mix (Roche, 04896866001). All the mRNA data were normalized to *Gapdh*.

### 2.6. Cell Culture

The H9C2 cardiomyocytes were purchased from the Cell Bank of the Chinese Academy of Sciences (Shanghai, China). All the cells were seeded in Dulbecco's modified Eagle's medium (DMEM) (GIBCO, C11995), supplemented with 10% fetal bovine serum (FBS) (Gibco, 10099), 100 U/ml penicillin, and 100 *μ*g/ml streptomycin (Gibco, 15140). Only cells below passage 10 were used in our study. Next, cells were divided into groups and given different stimuli. The five doses of GE were determined according to our previous studies [16, 17]. After being seeded onto 6-well for 48 h, H9C2 were starved for another 16 hours and then were treated with DOX (1 *μ*mol/l) or DOX+GE (1 *μ*mol/l, 10 *μ*mol/l, 25 *μ*mol/l, 50 *μ*mol/l, and 100 *μ*mol/l) for 12 hours. To confirm our hypothesis that AMPK*α* is involved in GE-induced cardiac protective effects, adenoviral vectors carrying AMPK*α*2 small hairpin RNAs (shAMPK*α*) or the scrambled shRNA were used to infect H9C2 cells at a MOI of 100 for 4 h. The source of shAMPK*α* and the scrambled shRNA was described in our previous studies [16, 17]. To detect cell viability, the cells were seeded onto 96-well plates and were treated with DOX or DOX+GE (100 *μ*mol/l) for 24 hours.

### 2.7. TUNEL Staining

TUNEL staining was performed as previously described [23]. Briefly, a TUNEL assay (Millipore, USA) was used according to the manufacturer's instructions. A fluorescence microscope (Olympus DX51) was used to evaluate apoptotic cells. Image-Pro Plus 6.0 was used to quantify all the images.

### 2.8. Measurement of Cardiac Troponin-I Levels

At three days after DOX injection, blood was collected from the retro-orbital plexus. Plasma was centrifuged for 10 min at 1000 g to obtain the supernatant. After that, a mouse cardiac troponin-I (cTnI) ELISA kit from Life Diagnostics Inc. (West Chester, PA) was used to detect the levels of troponin in mice.

### 2.9. Measurement of CK, LDH, GSH, Gpx, MDA, 4-HNE, and 3-NT Levels

At three days after DOX injection, blood samples were collected and centrifuged for plasma separation (1000× g, 10 minutes, 4°C). The levels of creatine kinase (CK) and lactate dehydrogenase (LDH) were detected using the commercial kits (Nanjing Jiancheng Bioengineering Institute, China) according to the instructions.

The heart tissues were placed in cold normal saline and homogenized. After that, the supernatant was obtained through centrifuging (10,000× g, 5 minutes, 4°C) to detect the glutathione (GSH), glutathione peroxidase (Gpx), malondialdehyde (MDA), 4-hydroxynonenal (4-HNE)-protein adducts, and 3-nitrotyrosine (3-NT) levels in heart tissues. 4-HNE and 3-NT ELISA Kits were provided by Abcam.

In vitro, the levels of LDH and MDA were also evaluated by corresponding kits and the treatment protocols were strictly carried out according to the manufacturer instructions.

### 2.10. Measurement of SOD and Caspase 3 Activities

The activities of total superoxide dismutase (SOD) and caspase 3 were assessed spectrophotometrically in the fresh heart samples and cell culture supernatants using commercial assay kits (Nanjing Jiancheng Bioengineering Institute, China) according to the provided instructions.

### 2.11. Measurement of Intracellular ROS

Intracellular ROS production was detected using 2,7-dichlorodihydrofluorescein diacetate (DCF-DA, Invitrogen) as an indicator according to a previous study [16]. In brief, H9c2 myocytes were cultured in 96-well plates and pretreated with GE and DOX for 2 hours. After that, the cells were incubated with DCFH-DA (10 *μ*M) for 60 min in 37°C. The fluorescence was quantified using a spectrofluorometer with an excitation wavelength of 488 nm and emission at 525 nm.

### 2.12. Statistical Analysis

Quantitative data were presented as mean ± standard deviation (SD). Comparisons between two groups were performed using Student's *t*-test. A one-way analysis of variance (ANOVA) followed by Tukey's post hoc test was used to analyze differences among groups. *P* < 0.05 was considered statistically significant.

## 3. Result

### 3.1. GE Improved Cardiac Function and Alleviated Cardiac Injury in Mice with DOX Injection

**Figure 1:**
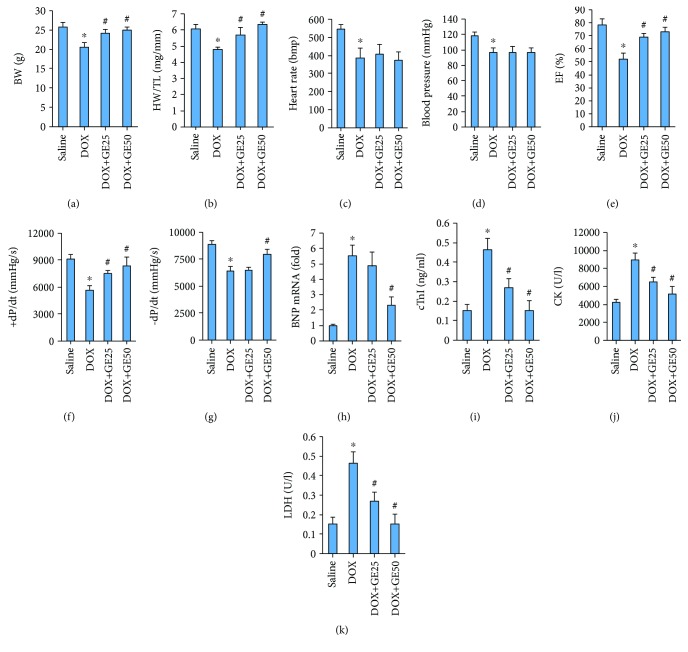
GE improved cardiac function and alleviated cardiac injury in mice with DOX treatment. (a) Body weight of four groups (*n* = 10). (b) Statistical results of the heart weight (HW)/tibia length (TL) (*n* = 10). (c) Heart rate of mice in four groups. (d) Maximum pressure. (e) Ejection fraction (EF) of mice (*n* = 10). (f, g) Hemodynamic analysis of mice with or without GE treatment (*n* = 10). (h) The mRNA expressions of hypertrophic markers (*n* = 10). (i) Plasma cardiac troponin-I (cTnI) levels. (j, k) Levels of creatine kinase (CK) and lactate dehydrogenase (LDH). Values represent the mean ± SD. ^∗^*P* < 0.05 versus saline, ^#^*P* < 0.05 versus DOX.

In the present study, 25 or 50 mg/kg of GE and 15 mg/kg of DOX were given according to our previous study [17, 23]. The injection of DOX obviously decreased body weight (BW), and GE at the dose of 25 or 50 mg/kg reduced weight loss during DOX treatment and restored body weight to the normal level (Figure 1(a)). Meanwhile, DOX also resulted in a decreased ratio of HW to TL, and conversely, GE could block this pathological decline (Figure 1(b)). DOX injection also led to a decrease in heart rate and blood pressure; however, there was no difference in the two between the DOX and DOX+GE groups (Figures 1(c) and 1(d)). Echocardiography and hemodynamics revealed that DOX treatment resulted in a reduction in ejection fraction (EF), maximal rate of the increase of left ventricular pressure (+dP/dt), and the maximal rate of the decrease of left ventricular pressure (−dP/dt) and GE significantly attenuated these pathological alterations in a dose-dependent manner (Figures 1(e)–1(g)). It also showed that 50 mg/kg of GE attenuated the level of brain natriuretic peptide (BNP) in mice with DOX injection (Figure 1(h)). Plasma cTnI is a well-established marker to reflect cardiac cell damage in patients with DOX. Therefore, we detected cTnI level in plasma of mice from four groups and found that cTnI concentrations were significantly higher in all DOX-treated mice compared with the saline group and mice from the DOX+GE25 and DOX+GE50 groups had relatively lower levels of cTnI (Figure 1(i)). CK and LDH levels in mice with DOX only were all obviously increased compared with the control groups; however, GE significantly decreased the CK and LDH levels (Figures 1(j) and 1(k)).

### 3.2. GE Protected against DOX-Induced Oxidative Damage *In Vivo*

**Figure 2:**
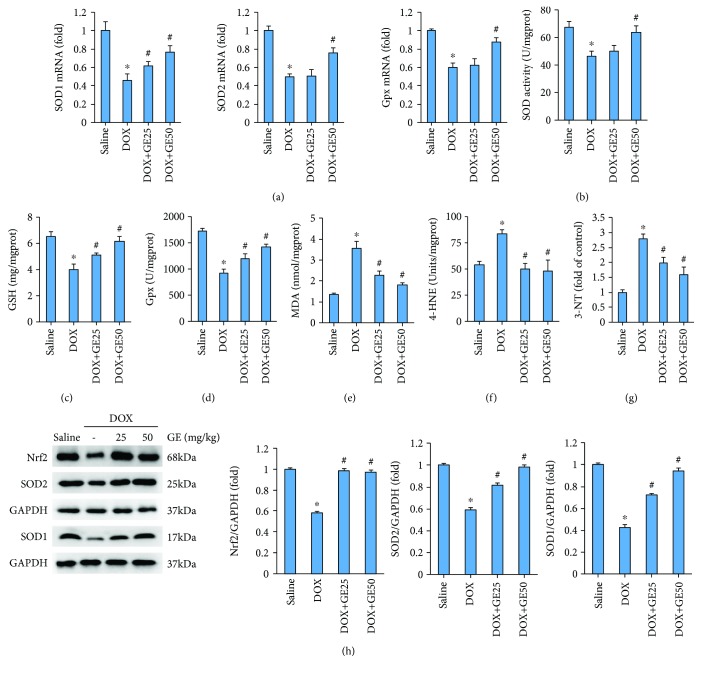
GE protected against DOX-induced oxidative damage in vivo. (a) The mRNA expressions of several antioxidant enzymes (*n* = 10). (b) Activity of superoxide dismutase (SOD) (*n* = 10). (c) Levels of glutathione (GSH) (*n* = 10). (d) Activity of glutathione peroxidase (Gpx) (*n* = 10). (e–g) Levels of malondialdehyde (MDA), 4-hydroxynonenal (4-HNE)-protein adducts, and 3-nitrotyrosine (3-NT) (*n* = 10). (h) Western blot and quantitative results in the indicated groups (*n* = 10). Values represent the mean ± SD. ^∗^*P* < 0.05 versus saline, ^#^*P* < 0.05 versus DOX.

To determine whether GE suppressed cardiac injury by inhibiting oxidative stress, we first examined levels of several antioxidant enzymes. As shown in Figure 2(a), DOX injection significantly downregulated the mRNA levels of SOD1, SOD2, and Gpx; however, the treatment of GE (50 mg/kg) significantly upregulated the mRNA levels of these antioxidant enzymes in DOX-treated mice. Next, we measured the activity of total SOD and found that SOD activity in mice from the DOX+GE50 group was enhanced compared with that in mice from the DOX group (Figure 2(b)). GE treatment also increased level of GSH and the activity of Gpx in mice with DOX injection (Figures 2(c) and 2(d)). Next, we detected the level of MDA in the hearts and found that the abnormal accumulation of MDA in DOX-treated hearts was largely reduced by GE treatment in a dose-dependent manner (Figure 2(e)). 4-HNE is a by-product of lipid peroxidation and is a stable marker of lipid peroxidation. 3-NT is a biomarker of protein oxidation produced upon the nitration of protein residues. The increased levels of 4-HNE and 3-NT could be observed in the hearts of mice with DOX only; however, after daily treatment of GE, these pathological upregulations were largely suppressed (Figures 2(f) and 2(g)). Western blot analyses were carried out to evaluate myocardial oxidative injury in mice with DOX injection. Nrf2 is an important transcription factor that regulates the cellular oxidative stress responses. Nrf2 can reduce the cell damage caused by reactive oxygen species. And the expression of Nrf2 was decreased in DOX-treated hearts, whereas the hearts from mice with DOX+GE showed increased level of Nrf2 (Figure 2(h)). Further detection of SOD1 and SOD2 expression also revealed that GE upregulated the expression of SOD1 and SOD2 in DOX-treated hearts (Figure 2(h)).

### 3.3. GE Attenuated DOX-Induced Cell Death *In Vivo*

**Figure 3:**
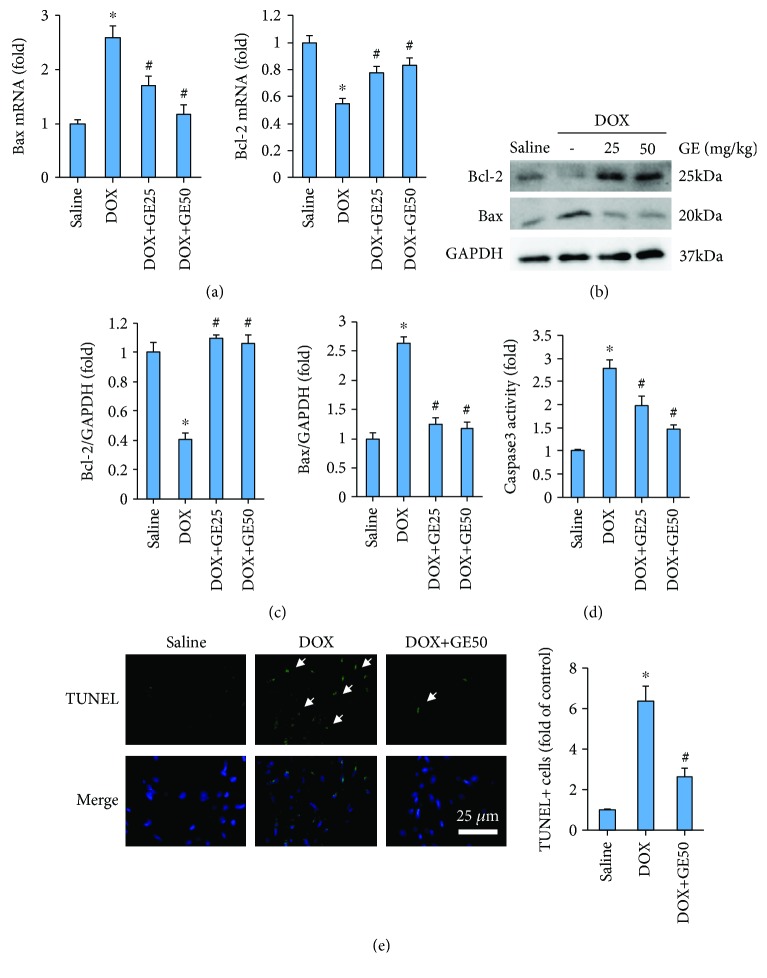
GE attenuated DOX-induced cell death in vivo. (a) The mRNA expressions of apoptosis markers (*n* = 10). (b, c) Western blot and quantitative results (*n* = 10). (d) Activity of caspase 3 of mice in four groups (*n* = 10). (e) Representative images of TUNEL and the quantitative results (*n* = 10). Arrows indicate TUNEL-positive cells. Values represent the mean ± SD. ^∗^*P* < 0.05 versus saline, ^#^*P* < 0.05 versus DOX.

Next, we investigated whether GE could attenuate DOX-induced cell apoptosis. DOX resulted in upregulation of Bax and downregulation of Bcl-2 in both mRNA level and protein level. However, after the intervention of GE, these pathological alterations were almost completely blocked (Figures 3(a)–3(c)). The activity of caspase 3 was also increased in the DOX group, which was markedly inhibited by GE in a dose-dependent manner (Figure 3(d)). The antiapoptotic effects of GE were further confirmed by TUNEL staining, which showed that GE significantly reduced TUNEL-positive cells (Figure 3(e)).

### 3.4. GE Inhibited DOX-Induced Toxicity *In Vitro*

**Figure 4:**
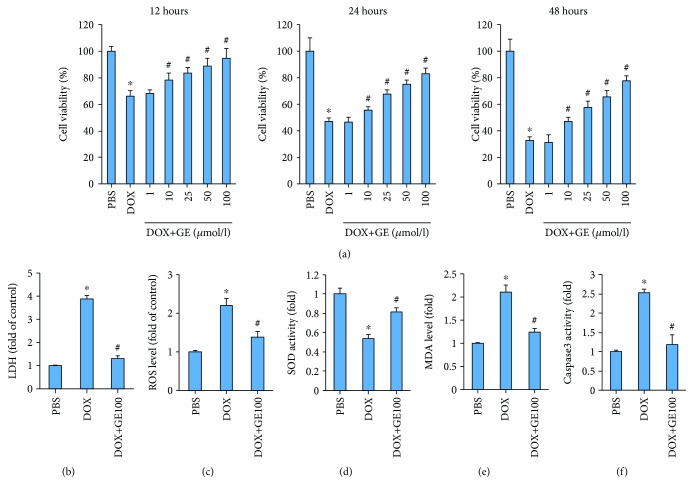
GE inhibited DOX-induced toxicity in vitro. (a) Cell viability after the treatment of DOX and GE with three different doses and durations (*n* = 3). (b) Levels of LDH in H9C2 cells (*n* = 3). (c) ROS level detected by DCF-DA in the indicated group (*n* = 3). (d) Activity of SOD in four groups (*n* = 3). (e) Levels of MDA (*n* = 3). (f) The activity of caspase 3 after GE (100 *μ*mol/l) for 12 h (*n* = 3). Values represent the mean ± SD. ^∗^*P* < 0.05 versus PBS, ^#^*P* < 0.05 versus DOX.

To further confirm the protective effects of GE in oxidative damage and apoptosis, we used H9C2 cardiomyocytes in vitro. H9C2 cells treated with escalating doses of GE exhibited increased cell viability in a dose-dependent manner at all three treatment durations (Figure 4(a)). Next, we detected the release of LDH to reflect cardiomyocyte injury and found that GE could significantly attenuate the content of LDH in DOX-treated cardiomyocytes (Figure 4(b)). DOX treatment resulted in ROS accumulation in cardiomyocytes, while GE decreased ROS levels in DOX-treated cells (Figure 4(c)). GE also significantly enhanced the activity of SOD and reduced the level of MDA in vitro (Figures 4(d) and 4(e)). GE significantly suppressed the activity of caspase 3 in DOX-treated cardiomyocytes (Figure 4(f)).

### 3.5. GE Activated AMPK*α* to Prevent DOX-Induced Toxicity *In Vitro*

**Figure 5:**
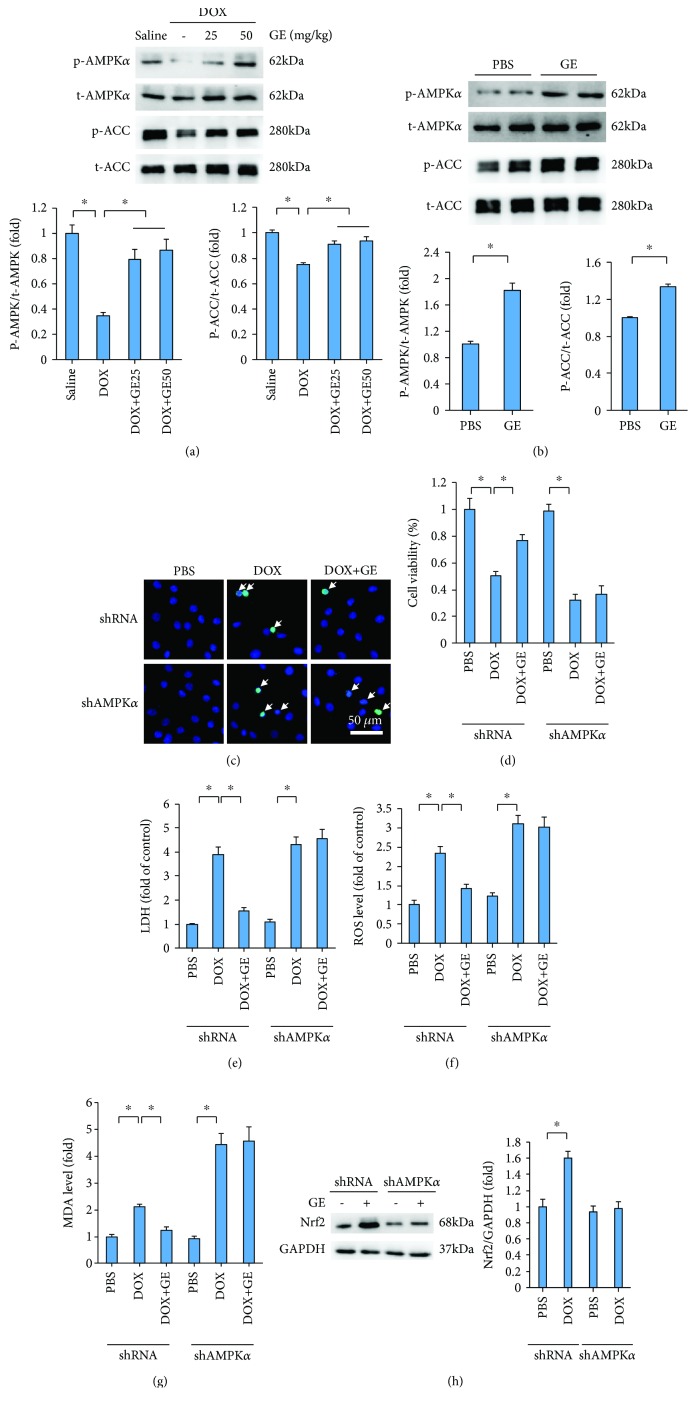
GE activated AMPK*α* to prevent DOX-induced toxicity in vitro. (a, b) Western blot and quantitative results in vivo (*n* = 3) and in vitro (*n* = 3). (c) Representative images of TUNEL and the quantitative results (*n* = 3). Arrows indicate TUNEL-positive cells. (d) Cell viability after GE (100 *μ*mol/l) for 12 h (*n* = 3). (e) LDH levels of four groups (*n* = 3). (f) ROS level detected by DCF-DA in the indicated group (*n* = 3). (g) Levels of MDA (*n* = 3). (h) Western blot and quantitative results of Nrf2 and GAPDH in H9C2 cells (*n* = 6). Values represent the mean ± SD. In (a), ^∗^*P* < 0.05 versus saline, ^#^*P* < 0.05 versus DOX; in (b), ^∗^*P* < 0.05 versus PBS. In others, ^∗^*P* < 0.05 versus DOX.

In previous investigation, we have indicated that GE could activate AMPK*α* to attenuate cardiac hypertrophy [17]. Therefore, we investigated in this study whether GE could attenuate cardiac injury induced by DOX via the activation of AMPK*α*. Western blot analysis revealed that phosphorylated AMPK*α* was remarkably decreased with the injection of DOX and restored after GE treatment in a dose-dependent manner (Figure 5(a)). Phosphorylation of ACC, a reflection of AMPK*α* activity, was also restored after GE treatment (Figure 5(a)). In agreement with this finding, we also found that GE increased expressions of p-AMPK*α* and p-ACC in vitro (Figure 5(b)). To verify the hypothesis that GE activated AMPK*α* to protect against DOX-related cardiac injury, we used shAMPK*α*2 to knock down AMPK*α* in H9C2 cells. The efficiency of shAMPK*α*2 has been detected in our previous study [17]. As shown in TUNEL staining, GE (100 *μ*mol/l) significantly inhibited DOX-induced cardiomyocyte apoptosis, and this effect of GE was abolished after AMPK*α* deficiency (Figure 5(c)). As shown in Figures 5(d) and 5(e), the protective effects of GE on cell viability and LDH release were completely offset after the infection of shAMPK*α*. GE also lost its protection against oxidative stress after AMPK*α* deficiency, as indicated by the elevated level of ROS and MDA (Figures 5(f) and 5(g)). Next, we detected whether the upregulation of Nrf2 by GE was also dependent on the activation of AMPK*α*. We found that and GE could increase Nrf2 expression even without any stimuli, and this effect was completely blocked by AMPK*α* deficiency (Figure 5(h)).

### 3.6. GE Lost Protection in Mice with AMPK*α* Deficiency

**Figure 6:**
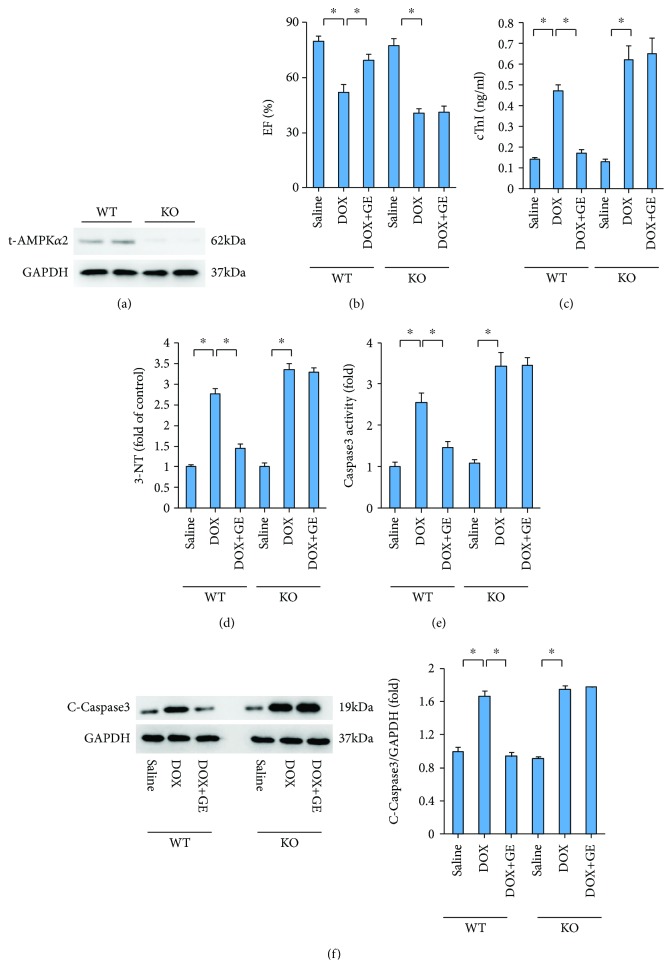
GE lost protection in mice with AMPK*α* deficiency. (a) The protein levels of t-AMPK*α*2 of C57 and AMPK*α*2 knockout mice (*n* = 10). (b) EF of mice (*n* = 10). (c, d) The levels of cTnI and 3-NT (*n* = 10) after the treatment of DOX and GE (50 mg/kg). (e) Activity of caspase 3 in the indicated group (*n* = 10). (f) The protein levels of caspase 3 and quantitative results (*n* = 10). Values represent the mean ± SD. ^∗^*P* < 0.05 versus DOX.

To further validate our finding, we used AMPK*α*2 knockout mice. The protein levels of t-AMPK*α*2 were detected by western blot in WT and AMPK*α*2 KO mice (Figure 6(a)). GE lost its protective effects against DOX-induced cardiac injury, as reflected by decreased EF and increased cTnI (Figures 6(b) and 6(c)). There was no difference between mice from the KO+DOX and KO+DOX+GE groups in 4-HNE level (Figure 6(d)). Further detection of C-Caspase3 expression and its activity revealed that AMPK*α* deficiency abolished the antiapoptosis effect of GE (Figures 6(e) and 6(f)).

## 4. Discussion

Here, we demonstrated for the first time that GE could improve cardiac function and alleviate cardiac injury induced by DOX. The major finding was that GE exerts antioxidative damage and antiapoptotic effects to attenuate DOX-induced cardiotoxicity via the activation of AMPK*α* from both in vivo and in vitro experiments. Moreover, AMPK*α* deficiency abolished GE-mediated cardiac protection against DOX-related cardiac injury. Our investigation provided a novel approach for the treatment of DOX-induced cardiac damage.

Oxidative stress is closely involved in the process of DOX-induced cardiac injury. DOX resulted in the accumulation of ROS, thus promoting the generation of by-product of lipid peroxidation via reacting with myocyte membranes [26] and ultimately leading to cardiac dysfunction. It has been reported that 4-HNE could directly impair cardiac contractile function at cardiac myocyte level [27]. In view of the damage caused by oxidative stress, adjustment of this redox balance might attenuate DOX-induced injury. The data in our lab demonstrated that GE decreased the level of 4-HNE and MDA in isoprenaline-related cardiomyopathy [28]. Therefore, we investigated whether GE could inhibit DOX-induced oxidative damage. In our study, DOX challenge resulted in increased levels of 4-HNE, 3-NT, and MDA, accompanied by downregulation of Nrf2 and expressions and activities of SOD. All of the above alterations were alleviated by GE, suggesting that GE may correct DOX-induced disturbed redox status. Excessive oxidative stress resulted in cell apoptosis. GE attenuated cardiomyocyte apoptosis in high-fat diet-induced cardiac injury [17]. Consistent with this report, we demonstrated that GE inhibited DOX-induced cell loss in vivo and in vitro. We also found that GE largely attenuated DOX-related cardiac injury, as indicated by the release of cTnI, and improved cardiac function in DOX-treated mice.

Another question arises, that is, how GE exerted its protective effect. Accumulating evidence has shown that AMPK*α* is an indispensable regulator of a wide variety of physiological events, especially in cardiovascular diseases [29]. Abundant evidences illustrated the close association between AMPK*α* and DOX-induced heart injury. It has been reported that phosphorylation of AMPK*α* protein level was slightly increased after DOX treatment [30–32]. However, there sounds a quite different voice. Other studies found that DOX significantly decreased AMPK*α* expression [8, 33, 34]. Consistent with these studies, we also found that DOX treatment significantly attenuates the expression and activity of AMPK*α* in vivo. Moreover, GE could attenuate DOX-induced cardiac dysfunction via the activation of AMPK*α* and AMPK*α* deficiency abolished the GE-mediated protective effects both in vivo and in vitro, indicating that improved cardiac function caused by GE was at least partly mediated by AMPK*α*. Our previous study found that activation of AMPK*α* could inhibit oxidative stress and cell apoptosis [19]. Therefore, we verify whether the protection against oxidative stress and cell death provided by GE was also mediated by AMPK*α*. The data in our study demonstrated that the upregulation of Nrf2 caused by GE was inhibited by the knockdown of AMPK*α* in vitro. GE lost its inhibitory effect on ROS accumulation and production of lipid peroxidation in shAMPK*α*-infected cells after DOX injection. Using AMPK knockout mice, we found that GE cannot provide any protection against the production of 4-HNE after AMPK deficiency. Cell viability assay, TUNEL staining, and caspase 3 activity detection suggested that GE lost its inhibitory effects on cardiomyocyte apoptosis. The finding indicated that GE protected against DOX-related oxidative injury and cell apoptosis via activating AMPK*α*.

GE has been widely used in Asia and has extensive pharmacological actions for the treatment. Inchin-ko-to, a “magic pill” for jaundice, is mainly composed of an analogue of GE and has been clinically used in China and Japan for a long time [35]. In our previous study, we also found that mice treated with GE (50 mg/kg) exhibited no obvious change in hepatic morphology [16]. However, the attenuation of DOX-caused systematic injury (body weight loss) should not be all attributed to cardiac benefit provided by GE treatment. GE may also reduce the damage in other organs, which needs further investigation. Considering the translational potential of these findings, it was of importance to confirm that GE does not compromise therapeutic DOX levels or promote tumor growth. It has been reported that GE has been shown to inhibit DNA synthesis of tumor cells and inhibit cell cycle progression [36, 37]. However, there was no report on the effect of GE on the metastasis of tumor.

Collectively, our present study demonstrated that GE could protect against DOX-induced cardiac toxicity, inhibiting oxidative stress and apoptosis of cardiomyocytes via the activation of AMPK*α*. In addition, our findings provide a promising therapeutic approach to treat chemotherapeutic agent-induced cardiotoxicity.

## Data Availability

The data used to support the findings of this study are available from the corresponding authors upon request.
